# Risk‐based breast cancer follow‐up stratified by age

**DOI:** 10.1002/cam4.1760

**Published:** 2018-09-11

**Authors:** Annemieke Witteveen, Jan W. M. Otten, Ingrid M. H. Vliegen, Sabine Siesling, Judith B. Timmer, Maarten J. IJzerman

**Affiliations:** ^1^ Department of Health Technology and Services Research University of Twente Enschede The Netherlands; ^2^ Department of Stochastic Operations Research University of Twente Enschede The Netherlands; ^3^ Department of Industrial Engineering and Innovation Sciences Eindhoven University of Technology Eindhoven The Netherlands; ^4^ Department of Research Netherlands Comprehensive Cancer Organisation (IKNL) Utrecht The Netherlands; ^5^ Faculty of Medicine Dentistry and Health Sciences School of Population and Global Health University of Melbourne and Victorian Comprehensive Cancer Centre Melbourne Victoria Australia

**Keywords:** breast cancer, follow‐up, locoregional recurrence, partially observable Markov decision process, second primary

## Abstract

Although personalization of cancer care is recommended, current follow‐up after the curative treatment of breast cancer is consensus‐based and not differentiated for base‐line risk. Every patient receives annual follow‐up for 5 years without taking into account the individual risk of recurrence. The aim of this study was to introduce personalized follow‐up schemes by stratifying for age. Using data from the Netherlands Cancer Registry of 37 230 patients with early breast cancer between 2003 and 2006, the risk of recurrence was determined for four age groups (<50, 50‐59, 60‐69, >70). Follow‐up was modeled with a discrete‐time partially observable Markov decision process. The decision to test for recurrences was made two times per year. Recurrences could be detected by mammography as well as by self‐detection. For all age groups, it was optimal to have more intensive follow‐up around the peak in recurrence risk in the second year after diagnosis. For the first age group (<50) with the highest risk, a slightly more intensive follow‐up with one extra visit was proposed compared to the current guideline recommendation. The other age groups were recommended less visits: four for ages 50‐59, three for 60‐69, and three for ≥70. With this model for risk‐based follow‐up, clinicians can make informed decisions and focus resources on patients with higher risk, while avoiding unnecessary and potentially harmful follow‐up visits for women with very low risks. The model can easily be extended to take into account more risk factors and provide even more personalized follow‐up schedules.

**Abbreviations**
B: belief
DCIS: ductal carcinoma in situ
LRR: locoregional recurrence
MDP: Markov decision process
M: mammography
NCR: Netherlands Cancer Registry
POMDP: partially observable Markov decision process
QALY: quality‐adjusted life year
SP: second primary

## 1 BACKGROUND

The incidence of breast cancer is rising.^1^ The number of new invasive breast cancer cases in the Netherlands is currently more than 14 000 per year, which accounts for over 28% of all cancer cases in women.^2^ Of the patients treated for primary invasive breast cancer, 4% will develop a locoregional recurrence (LRR) and almost 5% will be diagnosed with a second primary (SP) breast cancer in the 10 years following the primary diagnosis.^2^ In the Netherlands, patients are followed clinically for at least 5 years after primary treatment to detect recurrent disease early using annual mammography and improve survival.^3^, ^4^ Using regular follow‐up, 34% of all recurrences are found asymptomatically, justifying the follow‐up.^5^ However, frequency and duration are still debated, because half of the recurrences are actually found by self‐detection in between follow‐up visits.^5^ Although routine follow‐up can provide reassurance, it also induces anxiety and stress in patients. Additionally, there is disutility and unnecessary costs from false‐positive tests and subsequent invasive biopsies.^6^, ^7^ Because of these limitations, and because of an increasing population with breast cancer, there is a potential shortage in healthcare capacity. Consequently, a more personalized follow‐up, targeting intensive follow‐up to those at high risk for recurrence is a necessary approach to allocate scarce resources and to optimize detection.

Risk factors for recurrence are known and can be used to identify women for a more intensive follow‐up.^8^ However, in addition to risk of recurrence, progression of the disease and the decision whether or not to test as well as the health outcomes need to be modeled. Decision trees are often used for this, but are of limited use when modeling complex systems involving time. An alternative method to model health states and future events is Markov state‐transition modeling, where the transition from one state to another depends on transition probabilities.^9^ To capture processes with both transitions of health states by chance and decisions, Markov decision processes (MDPs) can be used. MDPs are a powerful tool for modeling screening decisions that are made sequentially over time.^10^

In this study, a partially observable MDP (POMDP) is implemented in order to account for the uncertainty of being in a certain state, in this case because of diagnostic uncertainty of mammography (ie, low sensitivity).^11^ The probabilities of an observation given a certain state are captured in an information matrix. The state space represents all possible states of the patient that are considered in the model. Based on the current belief state (a probability distribution of the state space), the performed test and the observation from this test, the new belief state can be obtained using Bayesian updating.^10^ More basic information on the state and belief space and solving of a POMDP can be found in the Supporting information. Ayvaci et al^12^ developed a MDP for optimal diagnostic decisions given a BIRADS score of a mammogram in the breast cancer screening setting. As partial observability was not taken into account, this represents a very simplified model of the screening system. Although other studies in the screening setting did include partial observability of the true health state, they did not take into account quality of life^13^ or self‐detection.^13^, ^14^ The aim of this study was to provide a comprehensive model of the breast cancer follow‐up setting with a clinical focus that takes into account the different methods of detection and uncertainty of the true health state and maximizes the total expected quality‐adjusted life years (QALYs). Other details about the POMDP modeling approach are published elsewhere.^15^

## 2 MATERIALS AND METHODS

A POMDP was developed to determine optimal follow‐up strategies based on the individual risk of a LRR or SP. Transition probabilities to populate the POMDP were obtained from large data set from the Netherlands Cancer Registry (NCR) and included a wide range of clinical and tumor‐specific predictors. Based on the underlying risk prediction model, the POMDP was designed to evaluate the influence of age on optimal follow‐up. Stratification by age was chosen, as age is currently used to determine the follow‐up policy after the standard 5 years of follow‐up.

### 2.1 Model formulation

To allow a more frequent and flexible follow‐up, decision epochs of 6 months were chosen instead of the regular annual follow‐up. A finite 5‐year horizon was used, as most of the LRRs are detected in the first 5 year following curative treatment.^8^ The decision to test was based on the present belief about the health state, as the true health state is unknown. This belief was based on an individual's set of risk factors as well as her test history. A biopsy was performed following a positive mammography and in case of self‐detection. We assumed that a biopsy was 100% sensitive and specific. After a biopsy confirmed diagnosis, women receive treatment and subsequently were moved to the absorbing treatment state implying they did not return in the model after treatment. The other absorbing state in the model was death.

**Figure 1:**
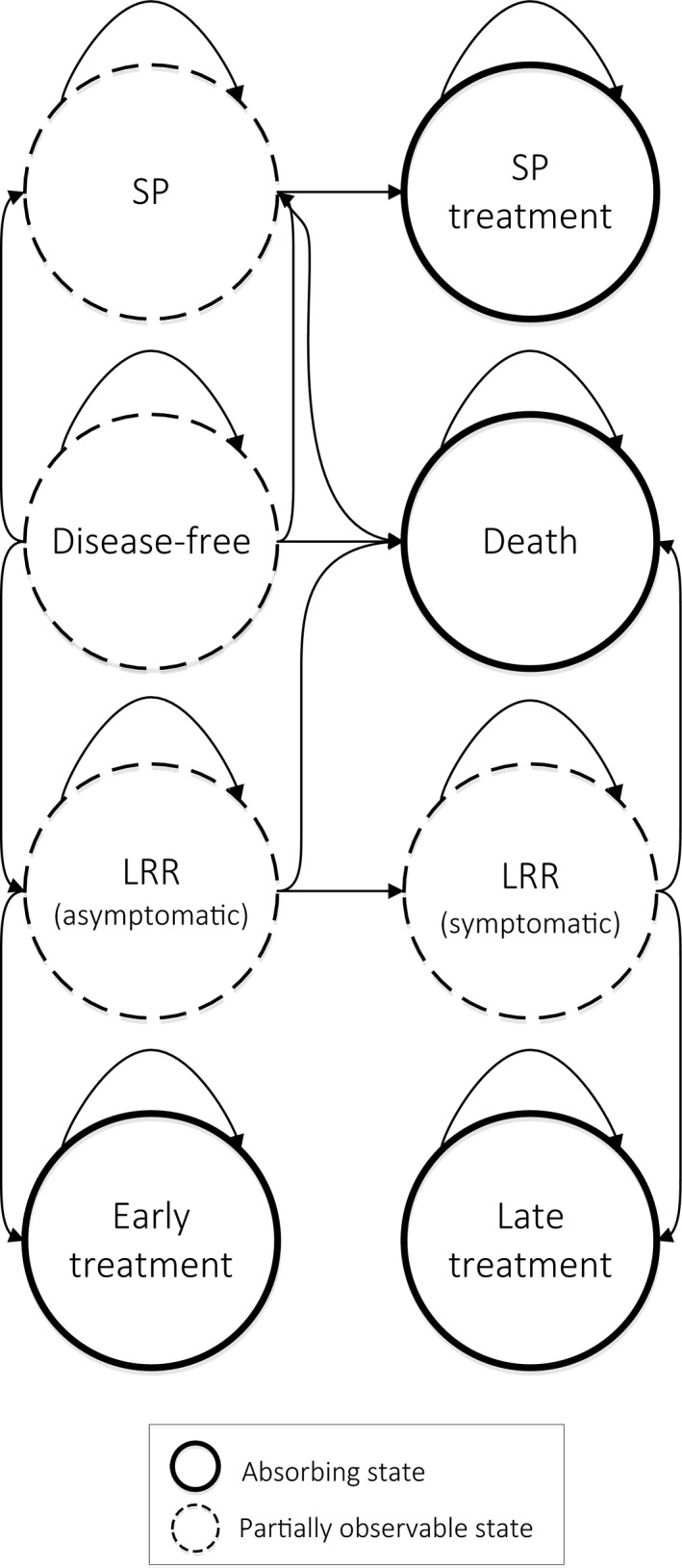
Health states incorporated in the model and their transitions. LRR, locoregional recurrence; SP, second primary

If a LRR was detected in an early phase, the treatment was less intensive and outcomes were better. During the follow‐up, women could remain in the disease‐free state or move to the SP tumor or LRR state. An asymptomatic LRR could be detected by mammography, while after progressing to the symptomatic LRR, detection by both mammography and self‐detection could take place. Also, women could move to the death state from every nonabsorbable state. For all the states and their transitions see Figure 1.

### 2.2 Model inputs

To define the transition probabilities from the disease‐free state to LRR and SP 37 230 women with primary invasive breast cancer without DM between 2003 and 2006 were selected from the NCR. Please be referred to the online materials for a complete overview of the patients included. Logistic regression was used to calculate probabilities for each half year conditional on not being diagnosed with recurrence in the previous period. More details on the modeling of the transition probabilities can be found in the paper by Witteveen et al.^8^ The probability that a woman died between two decision epochs depended on age and was obtained from Statistics Netherlands.^16^ To determine the value of the expected survival after diagnosis, the average age of the group was used (eg, a woman of 55 for the age group 50‐59).

The sensitivity and specificity of both mammography and self‐detection were obtained from Kolb et al^17^ from a study comparing the performance of mammography, physical examination, and ultrasound based on 11 130 women with 27 825 screening sessions. Unlike specificity, the sensitivity of testing depended on the true health state as there were multiple cancer states.

Rewards expressed in QALYs depended on the true health state, the actions, and the observations that were made. The half‐cycle correction method was used when patients died in between decision epochs: It assumes that half the possible QALYs were accrued during this period.^18^ The total or lump‐sum rewards were based on the life expectancy of a healthy patient. The expected remaining life years for patients in the different cancer states were calculated as a percentage of the expected remaining life years of a healthy patient. These percentages were based on the 10‐year survival rates after recurrence for the different recurrence states, which were estimated from the NCR.^2^ Disutilities associated with mammography and biopsy were subtracted from the initial state reward. Estimations of the disutility of a mammogram ranged from 0.25 to 1.75 days, so a disutility of 1 day was chosen.^12^, ^19^, ^20^ The disutility of a biopsy was set to an average of 3 weeks, as ranges estimated from 2 to 4 weeks.^12^, ^20^, ^21^ Note that a reward was given when a mammography resulted in early detection. An overview of all the values of the model inputs can be found in Table 1. A sensitivity analysis was performed to assess the changes in optimal policies when varying the growth rate and survival benefit after early detection for the age groups (lump‐sum reward).

**Table 1:** Model inputs and sources

Input	Value				Source
	<50 (26%)	50‐59 (28%)	60‐69 (23%)	≥70 (23%)	
Probability of death	0.00114	0.00322	0.00777	0.01949	16
Transition to SP	0.002588	0.002482	0.002482	0.001956	2
Probability of LRR per decision epoch^a^					2
1	0.13	0.08	0.07	0.14	
2	0.35	0.23	0.14	0.00	
3	0.51	0.28	0.24	0.36	
4	0.43	0.35	0.31	0.33	
5	0.36	0.25	0.18	0.27	
6	0.31	0.21	0.28	0.30	
7	0.28	0.23	0.24	0.25	
8	0.30	0.16	0.19	0.13	
9	0.27	0.15	0.27	0.16	
10	0.22	0.16	0.33	0.19	
Disutility of a mammogram	1 d	1 d	1 d	1 d	12, 19, 20
Disutility of a biopsy	21 d	21 d	21 d	21 d	21
Sensitivity mammography	0.580	0.827	0.827	0.827	17
Specificity mammography	0.988	0.988	0.988	0.988	17
Sensitivity self‐detection early LRR	0	0	0	0	
Sensitivity self‐detection	0.36	0.255	0.255	0.255	17
Specificity self‐detection	0.984	0.984	0.984	0.984	17
Reward based on life expectancy	39.44 y	30.10 y	21.35 y	13.37 y	2
Lump‐sum reward early LRR	0.86^a^ reward	0.85^a^ reward	0.85^a^ reward	0.90^a^ reward	2
Lump‐sum reward late LRR	0.69^a^ reward	0.68^a^ reward	0.70^a^ reward	0.70^a^ reward	2
Lump‐sum reward SP	0.80^a^ reward	0.80^a^ reward	0.80^a^ reward	0.85^a^ reward	2

LRR, locoregional recurrence; SP, Second Primary.

a Rounded values

### 2.3 Solving the optimality equations

To solve a MDP, the optimal value function of the state at each decision epoch is related to the value function of the next epoch. With a regular MDP, solving the value function optimality equation is done by iterating over the state space. However, since in this case the state is partially observable, it is required to consider the belief of being in that state, which constitutes a distribution of an infinite number of possibilities instead of one number. Because of characteristics of the POMDP model formulation, the value function is piecewise linear and convex, which can be represented by a finite number of vectors. The algorithm to solve the optimality equations of POMDPs was first described by Smallwood and Sondik^22^ and later simplified by Monahan^23^ and Lovejoy.^24^ First, the value functions for each action (wait or perform mammography) and decision epoch are calculated. This is iterated for the next decision epoch and with each iteration the number of vectors doubles. Subsequently, dominated vectors (which result in lower value) are discarded thus simplifying the problem. With the nondominated vectors, the value function is obtained for each belief state. In our example, the value refers to the maximization of the total expected QALYs. For more details and the exact equations, the reader is referred to Otten et al.^15^ After solving the equations, the economic impact of the risk‐based strategies was assessed using the average number of women starting follow‐up per year and the lower bound on the number of mammograms saved. Between the years 2003 and 2006, on average 9862 women started follow‐up after curative treatment of early invasive breast cancer in the Netherlands.

## 3 RESULTS

Life expectancies and lump‐sum rewards calculated as percentage of the healthy life expectancy for the four categories are presented in Table 1. The majority of the women did neither get a false‐ nor a true‐positive mammogram during follow‐up, as the risk of recurrent breast cancer was low and the specificity of mammography is good. The results are presented for the scenario assuming that no further test was positive, as a positive mammography influences the optimal schedule. Also, when follow‐up progressed the belief that a woman had recurrent breast cancer increased, even if there was a negative outcome of mammography or clinical examination. However, although the belief that a woman had a recurrence was lowered, it would not be zero as the test may have been imperfect. In addition, if a patient would test false‐positive, and additional testing would point out that a patient was in fact free of cancer, this would have brought the belief to zero and thereby change the follow‐up schedule.

**Figure 2:**
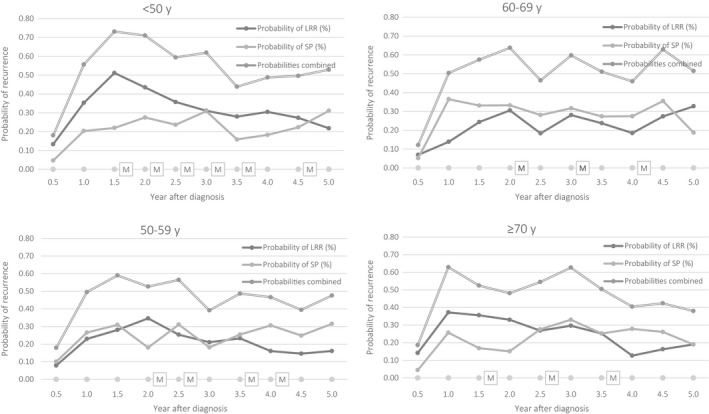
Optimal follow‐up schedules per age group. LRR, locoregional recurrence; SP, second primary; M, mammography advised

**Figure 3:**
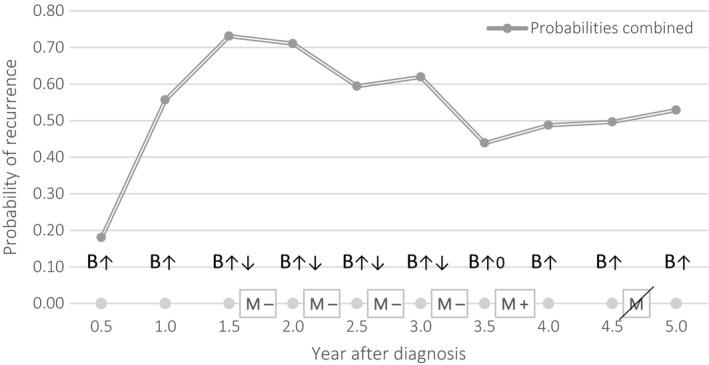
Change in optimal follow‐up schedules after a false‐positive and subsequent biopsy, as the belief of recurrent disease is brought back to 0 after confirmation that there is no recurrence by means of the biopsy. Arrows indicate the rising of the belief during the decision epoch and the lowering of the belief after a test is performed. B, belief; M−, mammography negative; M+, mammography positive; M, no mammography necessary (see M at 4.5 years in diagram)

Therefore, the optimal mammography schedules per age group at the start of the follow‐up, given that no positive test result was obtained are presented in Figure 2. A slightly more intensive follow‐up with one extra visit is proposed for the first age group (<50) with the highest risk. For the other age groups, we recommend less visits: four for ages 50‐59, three for 60‐69, and three for ≥70. As the risk of recurrent breast cancer was the highest around year two of follow‐up, the visits mostly concentrate in this period. The change in optimal schedules is exemplified in Figure 3. In this case, a woman had a false‐positive mammogram after 3.5 years, then underwent a biopsy confirming there was no recurrent disease resulting in a change of belief to zero so she could forgo the last mammogram at 4.5 years. Note that the belief also declined slightly even without mammography, as making no self‐detection during a decision epoch also lowered the belief state. As the optimal schedule can change during the follow‐up, the actual number of mammograms when using personalized schedules based on age will be lower and the numbers advised for the different age groups present an upper bound.

Using the average number of women starting follow‐up each year (N = 9862) combined with the age distribution and the upper bound on the number of mammograms, a lower bound on the number of mammograms saved was calculated (Table 2). The risk‐based policies resulted in over 9100 less mammograms (40 152 instead of 49 310) and a gain of 228 QALYs per cohort that starts follow‐up every year. As the costs of hospital mammography, including consultation with physical examination, are around €307^25^, the cost savings due less mammograms alone are estimated to be over €2.8 million per year. So besides gaining QALYs, these schedules also lead to at least 20% less follow‐up visits. On top of that would come the difference in treatment costs. When taking into account the women with ductal carcinoma in situ (DCIS) as well, the benefit and savings of risk‐based follow‐up would even be higher.

**Table 2:** Gain in QALYs when using risk‐based follow‐up

Age group	%	# of patients^a^	Gain in QALY/patient	Total gain in QALYs	Current policy (# of visits)	Advised risk‐based policy (# of visits)	Difference between policies	Total difference between policies
<50	26.24	2588	0.0424	109.7	5	6	+1	+2588
50‐59	28.45	2806	0.0201	56.4	5	4	−1	−2806
60‐69	22.60	2229	0.0093	20.7	5	3	−2	−4458
≥70	22.70	2239	0.0184	41.2	5	3	−2	−4478
Total	100	9862^a^	0.023	228.1	—	—	—	−9154

QALY, quality‐adjusted life year.

a (Based on) Average number of patients starting follow‐up per year during the years 2003‐2006.

The optimal policy was somewhat sensitive to the difference in lump‐sum rewards between an asymptomatically detected LRR and a symptomatically detected LRR as a percent of the healthy life expectancy (Table 3). It also seemed that the optimal result was quite sensitive to changes in the growth rate of a LRR: The number of mammograms changed when the LRR growth rate was multiplied by the factors 0.5, 1.5, 2, and 3 (Table 3). The effect of changes in the parameters was somewhat stronger for patients under 60 than for patients over 60 years.

**Table 3:** Sensitivity of the optimal number of visits

	Difference in reward between asymptomatic and symptomatic detected LRRs (percent point)							Growth rate LRR and transition to symptomatic phase, multiplied by				
	−6	−4	−2	0	2	4	6	×0.5	×1	×1.5	×2	×3
Age group												
<50	7	7	7	6	6	5	4	4	6	7	8	9
50‐59	4	4	4	4	3	3	2	2	4	4	6	7
60‐69	4	3	3	3	2	2	2	2	3	4	4	4
≥70	5	4	3	3	3	3	3	2	3	5	5	5

## 4 DISCUSSION

With a POMDP, optimal schedules were derived for four age groups considering the risk of recurrence, benefit of early detection and also disutility of (false‐positive) mammography and biopsies. It was optimal to have more intensive follow‐up around the peak in recurrence risk in the second year after diagnosis. A slightly more intensive follow‐up was proposed for the first age group with the highest risk, the other age groups were recommended less visits. We also found that the test history is of great influence on the optimal schedule. This risk‐based follow‐up would lead to a small increase in the total QALYs and a cost savings of over €2 800 000 per cohort starting follow‐up every year. However, the optimal number of follow‐up visits was sensitive to changes in the model inputs growth rate and life expectancy.

Within the age groups, there will still be heterogeneity in risk. To get toward truly personalized follow‐up models, more characteristics than only age need to be accounted for. Fortunately, the model can easily be extended to take into account more risk factors and provide even more personalized follow‐up schedules. Besides the risk of LRR, also SP risk was taken into account. This is especially important with longer follow‐up, as after 5 years, the risk of SP exceeds that of LRR and also while local control is increasing over time because of enhancements in treatment.^26^ As lifetime mortality is taken as an end point, the model itself introduces no lead‐time bias (overestimating the benefit of early detection by advancing the diagnosis^27^). And because there is no differentiation in the model between more or less aggressive tumor types, the results are averaged over all tumor types and there is no introduction of length bias (overestimating the benefit as screen‐detected tumors tend to be less aggressive and in earlier stages with better treatment outcomes^27^). Another strength of this study is that risks of LRR and SP were based on data from the nationwide population‐based NCR, providing generalizable estimates for the current breast cancer population.

The values used for the input parameters, however, are approximate. For example, the transition probabilities are derived from registry data from women undergoing annual follow‐up. The probabilities will therefore be slightly shifted in time as we only know the timing of diagnosis and not of onset. Other uncertainties are the transition from asymptomatic to symptomatic LRR and the reward for asymptomatic detected LRRs. Both were assessed for changes in optimal policies. Sensitivity analyses showed that the optimal policy is sensitive to changes in these parameters. The growth rate is hard to capture, and there will likely be fast‐growing tumors that we will be unable to detect asymptomatically even with intensive surveillance. The estimates of growth rates vary widely for primary breast cancer. Coumans et al^28^ found a range of volume doubling times between 2.0 and 11.2 months in 11 different articles. Estimates for recurrent breast cancer are missing all together. It is therefore important that studies to the natural history parameters of recurrent breast cancer are performed. The decision problem is also simplified by using only a two‐stage model for LRR, while in fact the growth of the tumor is continuous. With a continuous model, the progression would be portrayed less arbitrary and the lump‐sum rewards could be awarded more accurately. Also, the decision to test could be made at any time, instead of at predefined points in time.

To investigate the potential benefit of providing more intensive follow‐up, the decision to test was made on a biannual basis instead of annual. It is possible to look at smaller decision epochs, for example even in days, but very small epochs will not be clinically relevant, as it will not be possible or necessary to implement in clinical practice. The choice was made to use absorbent treatment states, as transition probabilities will have changed for individuals that have a history of recurrence. Sending them back into the model will provide unreliable estimates. Geurts et al^29^ found that although the risk of subsequent recurrence is high after the first recurrence, the absolute incidence remains low. And as almost half of those second recurrences are detected in the first year after the previous recurrence and more than 80% are DM, more intensive follow‐up for early detection subsequent recurrence is not likely to be (cost‐)effective.^29^

In summary, we demonstrated how follow‐up could be personalized based on the risk of recurrence for different age categories using a POMDP. With optimal risk‐based follow‐up schedules, clinicians will be able to make informed decisions and focus resources on patients with higher risk, while avoiding unnecessary and potentially harmful follow‐up visits for women with very low risks. However, there was uncertainty around the estimates which needs to be addressed in future modeling studies. The model can easily be extended to take into account more risk factors and provide even more personalized follow‐up schedules.

## CONFLICT OF INTEREST

The authors declare no conflict of interest.
