# Supplementary material for: Risk‐based breast cancer follow‐up stratified by age
Source: Cancer Med. 2018 Sep 11;7(10):5291–8. doi: 10.1002/cam4.1760 (PMC6198239; doi:10.1002/cam4.1760)
Supplement: Supplementary file 1 [file CAM4-7-5291-s001.pdf]

Supplement – Hypothetical example of a two stage POMDP

This is an example of a hypothetical two-stage POMDP where the stages are disease-free and not disease-free. The belief state is a probability distribution over the states, and all these possible probability distributions together form the belief space. Figure 1 below represents the belief space for an example two stage POMDP, with a probability distribution that sums up to 1. If the belief is 0, the patient is not believed to be in the disease-free state. As we only have two states, this would mean the patient is not disease-free. If the belief is 1, we know for certain that a patient is disease-free.

In our example we have two possible actions: performing a mammography and performing a biopsy. We will assume that a biopsy is a perfect test for diagnosing disease. Furthermore, we have two observations: a positive or a negative test. Let's say a patient has finished treatment and we are relatively sure she is disease-free. We take the blue dot in Figure 1 as our starting belief state. If we perform a mammography and it is negative (point C), we are even surer she is disease-free, but we are not 100% certain, as the sensitivity of mammography is not 100%. The same goes for a positive test. As the observations are probabilistic, each belief state associated with each observation has a probability associated with it. However, as we assume biopsy is perfect, a positive biopsy will bring us to point A, a belief of 0 for the disease-free state as we in fact know she is not disease-free. And vice versa for a negative test. In case of more states than two, the situation becomes more complex as the lines of the belief space will consist of hyperplanes.

Figure 1. Representation of the belief space of the disease-free state.

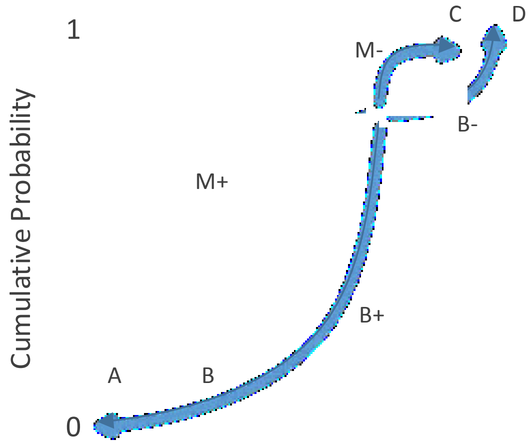

Abbreviations: M = mammography, B = biopsy.

The value you wish to maximize depends on the goal of your model. In this case it could be quality adjusted life years. In the example you can get a lower value from the disutility of testing itself, false-positives or finding cancer late, and a higher value when you find the cancer early. Without partial observability (in case of a MDP), the value could be represented by a table with one entry per state. But given the probability of being in a state, the value function becomes continuous, which gives problems when trying to solve it with value iteration. However, because of restrictions in the model formulation of POMDPs, the value function of a finite horizon is piecewise linear and convex: it can be represented with line segments (or hyperplanes) in a convex shape. The coordinates of the combined lines form a vector (Figure 2).

Figure 2. A. Example of a representation of the value function by vectors in a single time step. The upper surface of the line forms the value function. In this example it could be optimal not to test (a2) when you are fairly certain the patients is disease-free. **B.** If we start in belief state bs1 and take action a1, we end up in belief state bs2, where the next optimal action would be a2.

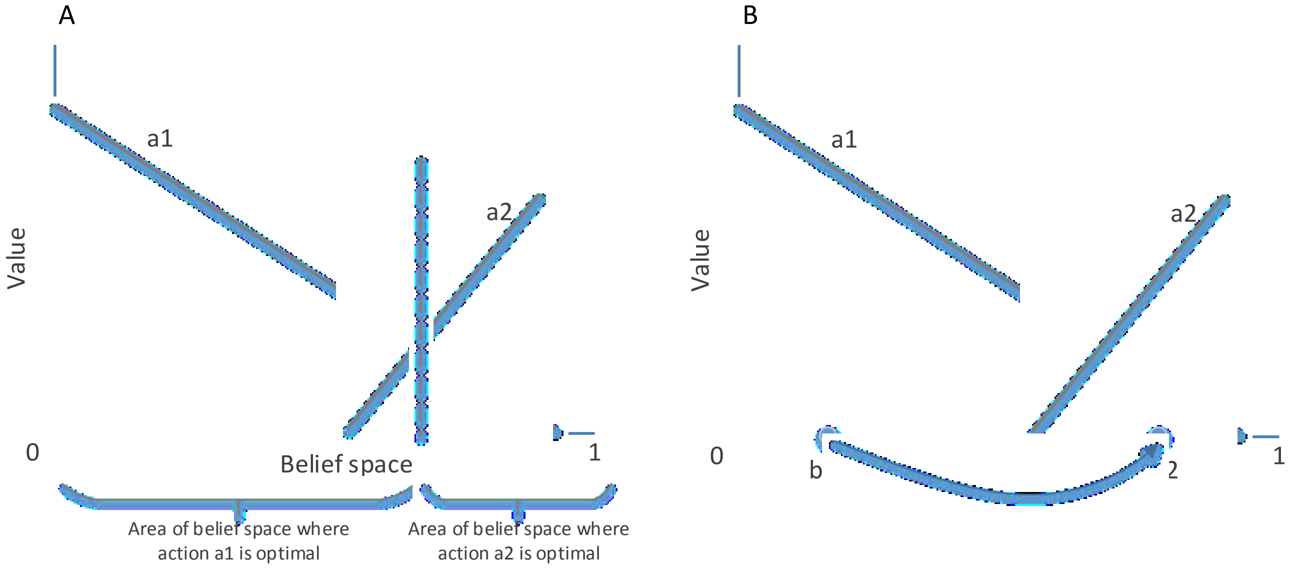

This leaves us with two vectors: one for the value and one which contains the probabilities of being in each of the possible states. If you take the dot product of these vectors, the value of a certain belief point is the maximum over these products. The next step will be to get the optimal action providing the highest value for each decision epoch. Using value iteration this not possible, as we cannot enumerate over the continuous states. For solving the problem algorithms are used to leave out the vectors that lead to non-optimal actions (specifics fall outside the scope of this example).
