# Supplementary material for: Risk‐based breast cancer follow‐up stratified by age
Source: Cancer Med. 2018 Sep 11;7(10):5291–8. doi: 10.1002/cam4.1760 (PMC6198239; doi:10.1002/cam4.1760)
Supplement: Supplementary file 2 [file CAM4-7-5291-s002.docx]

|  | | Total | | <50 | | 50-59 | | 60-69 | | ≥70 | |
| --- | --- | --- | --- | --- | --- | --- | --- | --- | --- | --- | --- |
|  |  | *N* | *%* | *N* | *%* | *N* | *%* | *N* | *%* | *N* | *%* |
| Total | | 37,230 | 100 | 9,771 | 26.2 | 10,593 | 28.5 | 8,415 | 22.6 | 8,451 | 22.7 |
| Histologic type | |  |  |  |  |  |  |  |  |  |  |
|  | Ductal | 29,547 | 79.4 | 8,064 | 82.5 | 8,506 | 80.3 | 6,628 | 78.8 | 6,349 | 75.1 |
|  | Lobular | 3,996 | 10.7 | 816 | 8.4 | 1,065 | 10.1 | 1,008 | 12.0 | 1,107 | 13.1 |
|  | Mixed | 1,550 | 4.2 | 409 | 4.2 | 471 | 4.4 | 333 | 4.0 | 337 | 4.0 |
|  | Other | 2,137 | 5.7 | 482 | 4.9 | 551 | 5.2 | 446 | 5.3 | 658 | 7.8 |
| Grade | |  |  |  |  |  |  |  |  |  |  |
|  | I | 7,620 | 20.5 | 1,419 | 14.5 | 2,415 | 22.8 | 1,949 | 23.2 | 1,837 | 21.7 |
|  | II | 15,574 | 41.8 | 3,708 | 37.9 | 4,266 | 40.3 | 3,719 | 44.2 | 3,881 | 45.9 |
|  | III | 11,467 | 30.8 | 4,036 | 41.3 | 3,165 | 29.9 | 2,169 | 25.8 | 2,097 | 24.8 |
|  | Unknown | 2,569 | 6.9 | 608 | 6.2 | 747 | 7.1 | 578 | 6.9 | 636 | 7.5 |
| Tumor size | |  |  |  |  |  |  |  |  |  |  |
|  | ≤2 cm | 22,584 | 60.7 | 5,291 | 54.2 | 6,963 | 65.7 | 5,854 | 69.6 | 4,476 | 53.0 |
|  | 2-5 cm | 13,226 | 35.5 | 4,018 | 41.1 | 3,257 | 30.7 | 2,333 | 27.7 | 3,618 | 42.8 |
|  | >5 cm | 1,092 | 2.9 | 356 | 3.6 | 273 | 2.6 | 168 | 2.0 | 295 | 3.5 |
|  | Unknown | 328 | 0.9 | 106 | 1.1 | 100 | 0.9 | 60 | 0.7 | 62 | 0.7 |
| Multifocal | |  |  |  |  |  |  |  |  |  |  |
|  | No | 23,206 | 62.3 | 5,821 | 59.6 | 6,555 | 61.9 | 5,401 | 64.2 | 5,429 | 64.2 |
|  | Yes | 4,160 | 11.2 | 1,424 | 14.6 | 1,200 | 11.3 | 796 | 9.5 | 740 | 8.8 |
|  | Unknown | 9,864 | 26.5 | 2,526 | 25.9 | 2,838 | 26.8 | 2,218 | 26.4 | 2,282 | 27.0 |
| Lymph node status | |  |  |  |  |  |  |  |  |  |  |
|  | Negative | 22,500 | 60.4 | 5,082 | 52.0 | 6,425 | 60.7 | 5,610 | 66.7 | 5,383 | 63.7 |
|  | 1-3 positive | 10,080 | 27.1 | 3,250 | 33.3 | 2,931 | 27.7 | 1,950 | 23.2 | 1,949 | 23.1 |
|  | >3 positive | 4,112 | 11.0 | 1,401 | 14.3 | 1,125 | 10.6 | 753 | 8.9 | 833 | 9.9 |
|  | Unknown | 538 | 1.4 | 38 | 0.4 | 112 | 1.1 | 102 | 1.2 | 286 | 3.4 |
| ER status | |  |  |  |  |  |  |  |  |  |  |
|  | Negative | 5406 | 14.5 | 1,825 | 18.7 | 1,572 | 14.8 | 1,044 | 12.4 | 965 | 11.4 |
|  | Positive | 23405 | 62.9 | 5,797 | 59.3 | 6,603 | 62.3 | 5,476 | 65.1 | 5,529 | 65.4 |
|  | Unknown | 8419 | 22.6 | 2,149 | 22.0 | 2,418 | 22.8 | 1,895 | 22.5 | 1,957 | 23.2 |
| PR status | |  |  |  |  |  |  |  |  |  |  |
|  | Negative | 9564 | 25.7 | 2,387 | 24.4 | 2,840 | 26.8 | 2,222 | 26.4 | 2,115 | 25.0 |
|  | Positive | 18855 | 50.6 | 5,201 | 53.2 | 5,190 | 49.0 | 4,166 | 49.5 | 4,298 | 50.9 |
|  | Unknown | 8811 | 23.7 | 2,183 | 22.3 | 2,563 | 24.2 | 2,027 | 24.1 | 2,038 | 24.1 |
| Her2-Neu status | |  |  |  |  |  |  |  |  |  |  |
|  | Negative | 13811 | 37.1 | 3,554 | 36.4 | 3,869 | 36.5 | 3,297 | 39.2 | 3,091 | 36.6 |
|  | Positive | 2401 | 6.4 | 826 | 8.5 | 730 | 6.9 | 479 | 5.7 | 366 | 4.3 |
|  | Unknown | 21018 | 56.5 | 5,391 | 55.2 | 5,994 | 56.6 | 4,639 | 55.1 | 4,994 | 59.1 |
| Type of surgery | |  |  |  |  |  |  |  |  |  |  |
|  | Breast conserving | 21028 | 56.5 | 5,400 | 55.3 | 6,756 | 63.8 | 5,441 | 64.7 | 3,431 | 40.6 |
|  | Non-breast conserving | 16202 | 43.5 | 4,371 | 44.7 | 3,837 | 36.2 | 2,974 | 35.3 | 5,020 | 59.4 |
| Axillary lymph node dissection | |  |  |  |  |  |  |  |  |  |  |
|  | No | 18369 | 49.3 | 4,242 | 43.4 | 5,403 | 51.0 | 4,603 | 54.7 | 4,121 | 48.8 |
|  | Yes | 18861 | 50.7 | 5,529 | 56.6 | 5,190 | 49.0 | 3,812 | 45.3 | 4,330 | 51.2 |
| Chemotherapy | |  |  |  |  |  |  |  |  |  |  |
|  | No | 23838 | 64.0 | 3,003 | 30.7 | 5,599 | 52.9 | 6,879 | 81.7 | 8,357 | 98.9 |
|  | Yes | 13392 | 36.0 | 6,768 | 69.3 | 4,994 | 47.1 | 1,536 | 18.3 | 94 | 1.1 |
| Radiotherapy | |  |  |  |  |  |  |  |  |  |  |
|  | No | 12735 | 34.2 | 3,025 | 31.0 | 2,932 | 27.7 | 2,403 | 28.6 | 4,375 | 51.8 |
|  | Yes | 24495 | 65.8 | 6,746 | 69.0 | 7,661 | 72.3 | 6,012 | 71.4 | 4,076 | 48.2 |
| Hormone therapy | |  |  |  |  |  |  |  |  |  |  |
|  | No | 21648 | 58.1 | 5,039 | 51.6 | 6,393 | 60.4 | 5,338 | 63.4 | 4,878 | 57.7 |
|  | Yes | 15582 | 41.9 | 4,732 | 48.4 | 4,200 | 39.6 | 3,077 | 36.6 | 3,573 | 42.3 |
